# Flow cytometric minimal residual disease measurement accounting for cytogenetics in children with non‐high‐risk acute lymphoblastic leukemia treated according to the ALL‐MB 2008 protocol

**DOI:** 10.1002/cam4.7172

**Published:** 2024-04-23

**Authors:** Alexander Popov, Guenter Henze, Grigory Tsaur, Oleg Budanov, Julia Roumiantseva, Mikhail Belevtsev, Tatiana Verzhbitskaya, Liudmila Movchan, Svetlana Lagoyko, Liudmila Zharikova, Yulia Olshanskaya, Tatiana Riger, Alena Valochnik, Natalia Miakova, Dmitry Litvinov, Olga Khlebnikova, Olga Streneva, Elena Stolyarova, Natalia Ponomareva, Galina Novichkova, Olga Aleinikova, Larisa Fechina, Alexander Karachunskiy

**Affiliations:** ^1^ National Research and Clinical Center for Pediatric Hematology, Oncology and Immunology Moscow Russian Federation; ^2^ Department of Pediatric Oncology Hematology Charité—Universitätsmedizin Berlin Berlin Germany; ^3^ Regional Children's Hospital Ekaterinburg Russian Federation; ^4^ Research Institute of Medical Cell Technologies Ekaterinburg Russian Federation; ^5^ Ural State Medical University Ekaterinburg Russian Federation; ^6^ Republican Scientific and Practical Center for Pediatric Oncology Hematology and Immunology Minsk Belarus; ^7^ Pirogov Russian National Research Medical University Moscow Russian Federation

**Keywords:** acute lymphoblastic leukemia, flow cytometry, genetic risk groups, minimal residual disease

## Abstract

**Background:**

Quantitative measurement of minimal residual disease (MRD) is the “gold standard” for estimating the response to therapy in childhood B‐cell precursor acute lymphoblastic leukemia (BCP‐ALL). Nevertheless, the speed of the MRD response differs for different cytogenetic subgroups. Here we present results of MRD measurement in children with BCP‐ALL, in terms of genetic subgroups with relation to clinically defined risk groups.

**Methods:**

A total of 485 children with non‐high‐risk BCP‐ALL with available cytogenetic data and MRD studied at the end‐of‐induction (EOI) by multicolor flow cytometry (MFC) were included. All patients were treated with standard‐risk (SR) of intermediate‐risk (ImR) regimens of “ALL‐MB 2008” reduced‐intensity protocol.

**Results and Discussion:**

Among all study group patients, 203 were found to have low‐risk cytogenetics (*ETV6*::*RUNX1* or high hyperdiploidy), while remaining 282 children were classified in intermediate cytogenetic risk group. For the patients with favorable and intermediate risk cytogenetics, the most significant thresholds for MFC‐MRD values were different: 0.03% and 0.04% respectively. Nevertheless, the most meaningful thresholds were different for clinically defined SR and ImR groups. For the SR group, irrespective to presence/absence of favorable genetic lesions, MFC‐MRD threshold of 0.1% was the most clinically valuable, although for ImR group the most informative thresholds were different in patients from low‐(0.03%) and intermediate (0.01%) cytogenetic risk groups.

**Conclusion:**

Our data show that combining clinical risk factors with MFC‐MRD measurement is the most useful tool for risk group stratification of children with BCP‐ALL in the reduced‐intensity protocols. However, this algorithm can be supplemented with cytogenetic data for part of the ImR group.

## 1 INTRODUCTION

Risk stratification in pediatric B‐cell precursor acute lymphocytic leukemia (BCP‐ALL) has traditionally been based on clinical characteristics such as age at diagnosis, WBC count, organomegaly, and CNS involvement.^1^ Later, parameters such as early response to glucocorticoids,^2^ bone marrow (BM) cytology during and at the end of induction (EOI)^3^ were introduced. Finally, genetic aberrations of leukemic cells^4^ and monitoring of minimal residual disease (MRD)^5^ were included and partially replaced the conventional clinical criteria. Currently, quantitative MRD measurement using multicolor flow cytometry (MFC) or PCR‐based techniques is the “gold standard” for estimating the effectiveness of therapy in childhood BCP‐ALL.^5^, ^6^ The speed of the MRD response differs for different cytogenetic and molecular genetic subgroups,^7^, ^8^ although the negative prognostic implications of slow MRD elimination in “favorable” groups are controversial.^7^, ^9^ However, the MRD measurement provides reliable stratification information in clinically defined risk groups, especially in the context of reduced‐intensity therapy.^10^, ^11^, ^12^

## 2 METHODS

Between February 2008 and November 2014, 3466 consecutive pediatric patients (aged 1 to 18 years) with ALL in Russia and Belarus were enrolled in the Moscow–Berlin group study ALL‐MB 2008 (NCT01953770). Patients with BCP‐ALL (*n* = 3044) were assigned to risk groups if they met the criteria listed in Table S1. Only patients from the standard risk (SR, *n* = 1702) and intermediate risk (ImR, *n* = 1105) groups were included. Children with high‐risk cytogenetics (*KMT2A* rearrangements, translocations t(17;19)(q22;p13)/*TCF3*::*HLF*, t(9;22)(q34;q11)/*BCR*::*ABL1* or intrachromosomal amplification of chromosome 21)^13^ were excluded. Other recently described high‐risk genetic aberrations (*PAX5*alt, *MEF2D*‐r, etc.) were not routinely assessed in all patients during the study period. The treatment design is shown in Figure S1. The treatment plan for SR and ImR has already been described in detail previously.^12^, ^14^ Briefly, all patients received induction therapy, followed by 3 cycles of consolidation and maintenance therapy (Figure S1). For logistical reasons, the MFC‐MRD pilot study was conducted in facilities attached to the MFC laboratories of the Moscow–Berlin group Flow‐network.^15^ A total of 485 patients with available cytogenetic data and BM samples for MFC‐MRD monitoring obtained at the EOI (Day 36) were included. The recurrence rate in the MRD study group was not different from that of the remaining 2309 patients of the ALL‐MB 2008 cooperative study with similar characteristics who did not participate in the MFC‐MRD study (Figure S2). Despite slightly different EFS (due to higher treatment‐related mortality in patients not screened for MFC‐MRD), the MFC‐MRD data were considered representative of all patients in the ALL‐MB 2008 study because the main consequence of slow MRD response is the development of relapse. Based on the cytogenetic data, patients were classified into low (high hyperdiploidy (HeH) and translocation t(12;21)(p13;q22)/*ETV6*::*RUNX1* ^13^) and intermediate (all others) cytogenetic risk groups.^13^ MRD was evaluated using a 6–9 color MFC in three laboratories using a well‐harmonized approach.^15^, ^16^ All laboratories use the same MFC methodology based on standard analyzes and had participated in the AIEOP‐BFM‐QA system^17^ and group‐internal proficiency tests.^15^ MRD negativity was defined as <0.01%.^18^, ^19^, ^20^ In addition to traditional threshold levels (TL) (0.01%, 0.1%, etc.), MFC‐MRD values were also categorized by quantitative TLs, with ROC analysis^21^ providing the best predictor of relapse. Event‐free survival (EFS) was defined as the time from diagnosis to the first event that is, non‐response, relapse, death from any cause, or second malignancy, whichever comes first. Observation periods were censored at the time of last contact if no events were reported. EFS curves were generated using the Kaplan–Meier method and standard errors were calculated according to Greenwood. Differences in outcome between groups were compared using the log‐rank test. Cumulative incidence of relapse (CIR) curves were estimated adjusting for competing risks of the other pertinent events and compared by Gray's test. All tests were two sided. Analyzes were performed using R‐statistics v3.4.2.

## 3 RESULTS AND DISCUSSION

**FIGURE 1:**
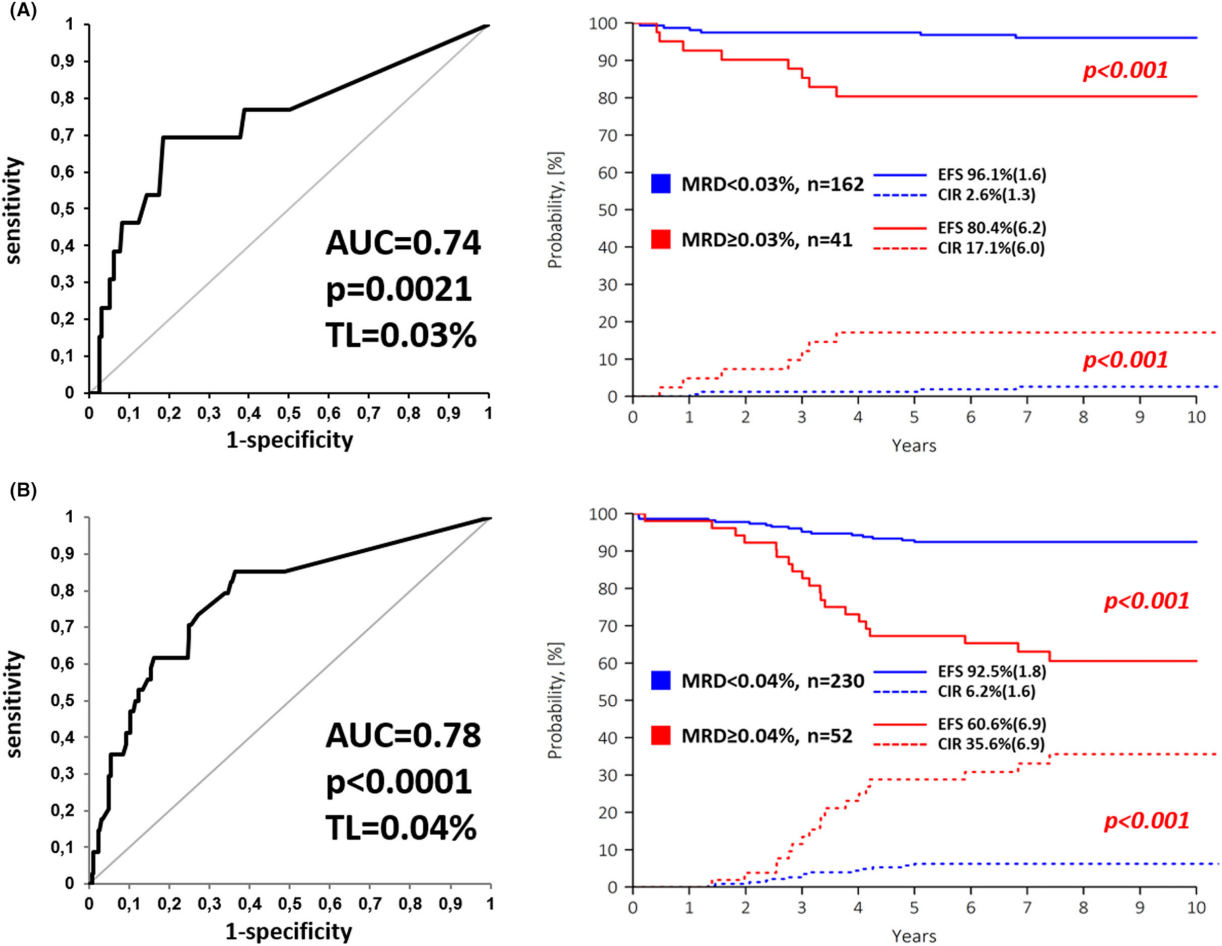
Definition and implementation of the exact threshold levels (TL) of MFC‐MRD values at EOI in patients with favorable cytogenetics (A) and in patients with intermediate cytogenetic risk (B). The diagrams on the left show the ROC curve with its most important features (area under the curve (AUC), *p*‐value and exact TL), which are the most meaningful parameters for predicting relapse. The graphs on the right show the event‐free survival (EFS, solid lines) and the cumulative incidence of relapse (CIR, dashed lines) as a function of the respective MFC‐MRD values according to the estimated TL. The standard errors are given in brackets.

Of the 485 patients included, 91 (18.8%) were diagnosed with HeH and 112 (23.1%) were *ETV6*::*RUNX1‐*positive. In this low cytogenetic risk group (*n* = 203), qualitative MFC‐MRD‐positivity at EOI did not allow unequivocal identification of a poor outcome patient. However, a quantitative threshold of 0.1% resulted in more accurate discrimination of slow responding patients with high relapse rates (Table S2A). ROC analysis revealed a TL of 0.03% (Figure 1A), which was the most representative for the low cytogenetic risk group (Table S2A, Figure 1A). The remaining 282 patients (58.2%) lacked either low‐ or high‐risk genetics and were therefore classified as intermediate genetic risk. For them, both conventional TLs of 0.01% and 0.1% as well as ROC‐defined TL of 0.04% (Figure 1B) were instructive for distinguishing patients with poorer and better outcomes (Table S2B).

**FIGURE 2:**
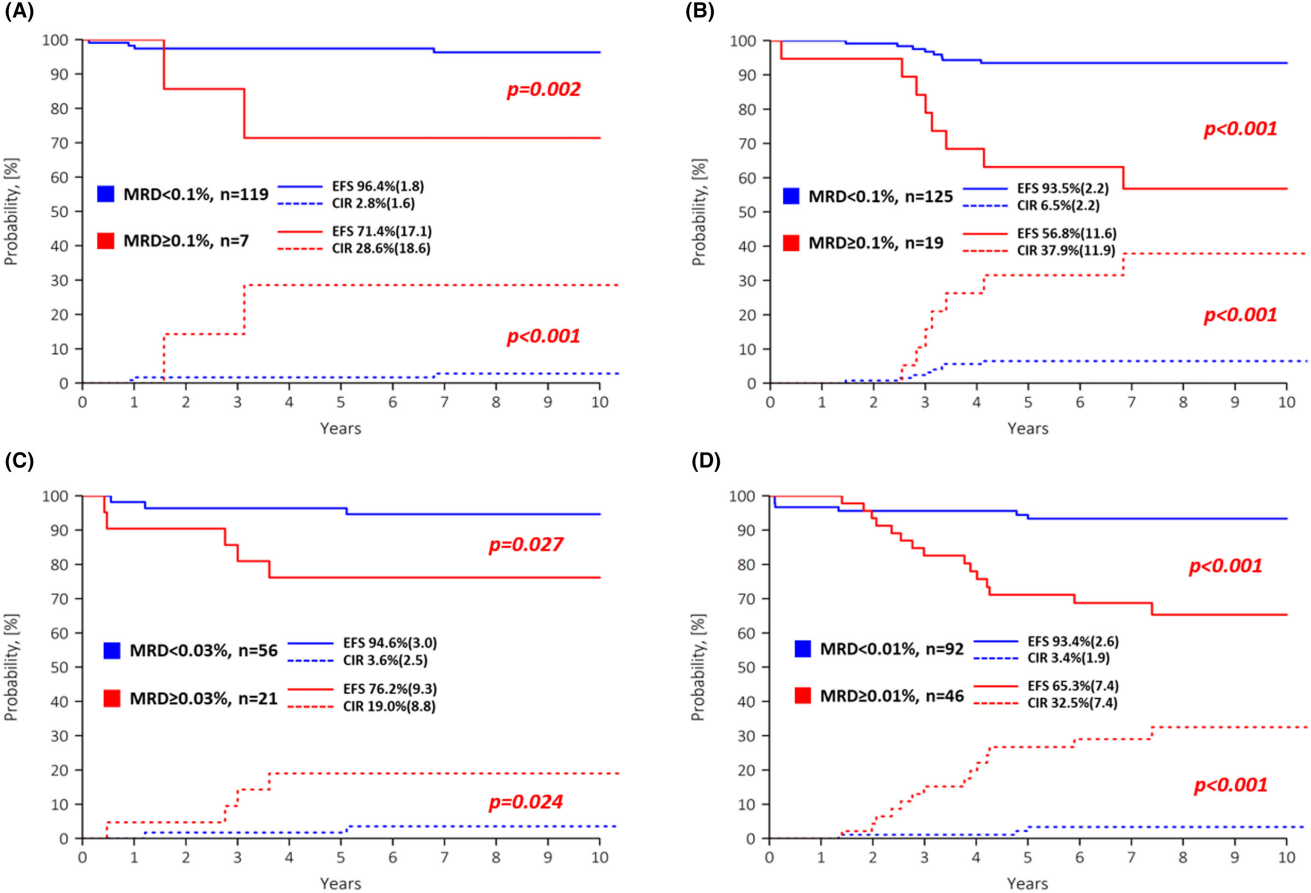
Event‐free survival (EFS, solid lines) and cumulative incidence of relapses (CIR, dashed lines) according to the best matching MFC‐MRD threshold values in patients with clinically defined standard risk (SR) and intermediate risk (ImR) groups (upper and lower row respectively). Panels (A) and (C) show results in patients with low risk cytogenetics and panels (B**)** and (D**)**—in patients with intermediate risk cytogenetics. Standard errors are given in brackets.

The most meaningful thresholds were different for clinically defined SR and ImR groups. As is known for the entire SR group, in patients with favorable cytogenetics, 0.1% was most effective for predicting recurrence (Table S2C, Figure 2A). In children with ImR cytogenetics who were assigned to the SR group at presentation, there was a clear distinction between children with different outcomes using all three TLs (Table S2E), although 0.1% was again the most conclusive (Figure 2B). In the ImR group, the situation was the same. For children with favorable cytogenetics, neither 0.1% nor the more conventional 0.01% were sufficiently discriminatory (Table S2D); only application of the ROC‐defined TL of 0.03% resulted in a clear distinction between fast and slow responders (Figure 2C). For ImR patients with intermediate cytogenetic risk, all three TLs were discriminatory (Table S2F), although the choice between them was not absolutely clear.

As previously described,^22^ the rationale for using MRD in the ALL‐MB 2008 reduced‐intensity protocol is to start with low‐ or moderate‐intensity treatment based on clinical risk factors. At the EOI, MFC‐MRD is used to assess response to therapy. The large group of those who respond well will continue with this therapy, and only for those who respond slowly should more intensive chemotherapy and/or immunotherapy be considered. The best TL is therefore the TL that makes it possible to identify as many “fast responders” as possible while maintaining their good prognosis^12^, ^14^ and to adapt subsequent therapy only for a few “slow responders.” According to this paradigm, for patients with low‐risk cytogenetics, the most meaningful TL appears to be 0.1% if they meet the criteria for SR group assignment, as previously indicated for the entire SR group.^12^, ^23^ If, on the other hand, they are assigned to the ImR by initial presentation parameters, the more precise TL of 0.03% should be used. For patients at intermediate cytogenetic risk, the final solution is similar. In children initially stratified into the SR group, all TLs used showed more or less equal results for the fast responding group, although the conventional SR group TL of 0.1% identified 87% of these “fast responding” individuals, versus 61% and 80% for the TLs of 0.01% and 0.04%, respectively (Table S2E). In contrast, for the patients in the clinically ImR group, a previously defined TL of 0.01%^14^ seems to be the most accurate (Figure 2D), even better than the ROC‐defined TL of 0.04%, since only patients with MFC‐MRD <0.01% showed a really good result with an acceptable recurrence rate (Table S2F).

Therefore, even accounting for cytogenetics, the combination of initial clinical SR criteria and MFC‐MRD at an EOI below 0.1% is still the best method for identifying children who can be successfully treated with low‐intensity chemotherapy. The ImR group also has the more precise ROC‐defined TLs, which are different for different genetic groups, although for the majority of children with ImR at first presentation (64.2% of children from the ImR group also have ImR cytogenetics) the generally defined TL of 0.01% is most applicable. For a significant proportion of patients with favorable cytogenetics in the ImR group, the more accurate TL of 0.03% for MFC‐MRD at EOI can be used.

There are currently several established genetic subgroups in pediatric BCP‐ALL.^4^ For these relatively new genetic subtypes, the role of MRD surveillance is also different.^7^, ^8^, ^9^, ^24^ Many of these new genetic groups are very rare or associated with very poor outcomes and therefore only affect the intermediate genetic risk group. Nevertheless, our concept of starting the therapy with reduced intensity from the beginning, taking into account clinical parameters combined with the MFC‐MRD measurement at the EOI, seems to be very effective even without the results of sophisticated genetic tests. Considering initial low‐risk parameters and a relatively high threshold of 0.1% for MFC‐MRD at the EOI, more than half of pediatric patients with BCP‐ALL can be identified who can be cured with low‐intensity therapy regardless of cytogenetic findings (except: high‐risk cytogenetics). On the other hand, the value of the MFC‐MRD for the post‐induction stratification of patients with initial ImR features is significantly dependent on their cytogenetic constellation.

## 4 CONCLUSION

Our data show that combining initial clinical risk factors with a single‐point MFC‐MRD measurement is the most useful tool for risk group stratification of children with BCP‐ALL in the context of reduced‐intensity protocols. However, this algorithm can be supplemented with cytogenetic data for part of the ImR group.

## AUTHOR CONTRIBUTIONS

**Alexander Popov:** Conceptualization (lead); data curation (lead); investigation (lead); methodology (lead); writing – original draft (lead); writing – review and editing (lead). **Guenter Henze:** Conceptualization (lead); data curation (equal); investigation (lead); writing – original draft (lead); writing – review and editing (lead). **Grigory Tsaur:** Conceptualization (lead); data curation (equal); investigation (equal); methodology (lead); writing – review and editing (equal). **Oleg Budanov:** Data curation (lead); investigation (lead); writing – review and editing (equal). **Julia Roumiantseva:** Conceptualization (equal); data curation (equal); writing – review and editing (equal). **Mikhail Belevtsev:** Investigation (equal); methodology (equal); writing – review and editing (equal). **Tatiana Verzhbitskaya:** Investigation (equal); methodology (equal); writing – review and editing (equal). **Liudmila Movchan:** Investigation (equal); methodology (equal); writing – review and editing (equal). **Svetlana Lagoyko:** Data curation (equal); writing – review and editing (equal). **Liudmila Zharikova:** Data curation (equal); writing – review and editing (equal). **Yulia Olshanskaya:** Data curation (equal); methodology (equal); writing – review and editing (equal). **Tatiana Riger:** Methodology (equal); writing – review and editing (equal). **Alena Valochnik:** Data curation (equal); methodology (equal); writing – review and editing (equal). **Natalia Miakova:** Conceptualization (equal); investigation (equal); project administration (equal); writing – review and editing (equal). **Dmitry Litvinov:** Investigation (equal); project administration (equal); writing – review and editing (equal). **Olga Khlebnikova:** Data curation (equal); writing – review and editing (equal). **Olga Streneva:** Data curation (equal); writing – review and editing (equal). **Elena Stolyarova:** Data curation (equal); writing – review and editing (equal). **Natalia Ponomareva:** Data curation (equal); writing – review and editing (equal). **Galina Novichkova:** Conceptualization (equal); data curation (equal); supervision (equal); writing – review and editing (equal). **Olga Aleinikova:** Conceptualization (equal); project administration (equal); supervision (equal); writing – review and editing (equal). **Larisa Fechina:** Conceptualization (equal); project administration (equal); supervision (equal); writing – review and editing (equal). **Alexander Karachunskiy:** Conceptualization (equal); investigation (equal); project administration (equal); supervision (equal); writing – review and editing (equal).

## FUNDING INFORMATION

This research did not receive any specific grant from funding agencies in the public, commercial, or not‐for‐profit sectors.

## CONFLICT OF INTEREST STATEMENT

The authors have no conflict of interest to declare.

## ETHICS STATEMENT

The study was approved by the Ethics Committee of the Dmitry Rogachev National Medical Research Center of Pediatric Hematology, Oncology and Immunology.

## PATIENT CONSENT STATEMENT

Informed consent for the collection and investigation of samples was obtained from patients' parents or legal guardians.

## CLINICAL TRIALS REGISTRATION

Moscow‐Berlin group study ALL‐MB 2008 trial is registered on clinicaltrials.gov with a reference number NCT01953770.

## Supporting information


Data S1.

## Data Availability

The datasets generated during and/or analyzed during the current study are available from the corresponding author on reasonable request.
