# Supplementary material for: Flow cytometric minimal residual disease measurement accounting for cytogenetics in children with non‐high‐risk acute lymphoblastic leukemia treated according to the ALL‐MB 2008 protocol
Source: Cancer Med. 2024 Apr 23;13(8):e7172. doi: 10.1002/cam4.7172 (PMC11036069; doi:10.1002/cam4.7172)
Supplement: Supplementary file 1 — Data S1. [file CAM4-13-e7172-s001.docx]

**Table S1.** ALL-MB 2008 risk stratification criteria

| Standard risk group | Intermediate risk group | High risk group |
| --- | --- | --- |
| all criteria must be met  BCP-ALL  initial WBC count <30×10^9^/L  spleen <4 cm below costal margin  no CNS3  no high-risk criteria | at least one criteria must be met  T-cell ALL  initial WBC count ≥30×10^9^/L  spleen ≥4 cm below costal margin  CNS-leukemia (CNS3)  and (obligatory) no high-risk criteria | at least one criteria must be met  BCP-ALL with initial WBC count ≥100×10^9^/L  t(4;11)(q21;q23)  t(9;22)(q34;q11)  no remission at day 36 |

**Table S2.** Event-free survival (EFS) and cumulative incidence of relapse (CIR) as a function of MFC-MRD values at EOI in relation to the conventional thresholds of 0.01% (top line of each panel), 0.1% (middle line of each panel) and the thresholds specifically defined for each group by ROC analysis (bottom line of each panel). Panel **A** shows data for patients with favorable genetics (low cytogenetic risk); Panel **B** shows data for children with intermediate cytogenetic risk; Panels **C** and **D** show patients with favorable genetics, divided into SR and ImR groups, respectively; Panels **E** and **F** show patients with intermediate risk cytogenetics who are categorized in SR or ImR groups. Standard errors (SE) are given in parentheses

| **Panel A** | | | | | | |
| --- | --- | --- | --- | --- | --- | --- |
| **MRD** | **n** | **relapses** | **EFS(SE)** | **p** | **CIR(SE)** | **p** |
|  | | | | | | |
| **<0.01%** | **125** | **4** | **95.1(2.0)** | **0.190** | **3.3(1.7)** | **0.070** |
| **≥0.01%** | **78** | **7** | **89.7(3.4)** |  | **9.0(3.3)** |  |
|  | | | | | | |
|  | | | | | | |
| **<0.1%** | **187** | **8** | **93.9(1.8)** | **0.083** | **4.5(1.5)** | **0.016** |
| **≥0.1%** | **16** | **3** | **81.2(9.8)** |  | **18.8(10.1)** |  |
|  | | | | | | |
|  | | | | | | |
| **<0.03%** | **162** | **4** | **96.1(1.6)** | **<0.001** | **2.6(1.3)** | **<0.001** |
| **≥0.03%** | **41** | **7** | **80.4(6.2)** |  | **17.1(6.0)** |  |
|  | | | | | | |
| **Panel B** | | | | | | |
| **MRD** | **n** | **relapses** | **EFS(SE)** | **p** | **CIR(SE)** | **p** |
|  | | | | | | |
| **<0.01%** | **180** | **7** | **94.4(1.7)** | **<0.001** | **4.0(1.5)** | **<0.001** |
| **≥0.01%** | **102** | **25** | **72.0(4.7)** |  | **26.0(4.6)** |  |
|  | | | | | | |
|  | | | | | | |
| **<0.1%** | **247** | **18** | **90.9(1.8)** | **<0.001** | **7.4(1.7)** | **<0.001** |
| **≥0.1%** | **35** | **14** | **55.6(8.7)** |  | **41.6(8.8)** |  |
|  | | | | | | |
|  | | | | | | |
| **<0.04%** | **230** | **14** | **92.5(1.8)** | **<0.001** | **6.2(1.6)** | **<0.001** |
| **≥0.04%** | **52** | **18** | **60.6(6.9)** |  | **35.6(6.9)** |  |
|  | | | | | | |
| **Panel C** | | | | | | |
| **MRD** | **n** | **relapses** | **EFS(SE)** | **p** | **CIR(SE)** | **p** |
|  | | | | | | |
| **<0.01%** | **78** | **2** | **95.9(2.3)** | **0.505** | **2.8(2.0)** | **0.272** |
| **≥0.01%** | **48** | **3** | **93.7(3.5)** |  | **6.2(3.5)** |  |
|  | | | | | | |
|  | | | | | | |
| **<0.1%** | **119** | **3** | **96.4(1.8)** | **0.002** | **2.8(1.6)** | **<0.001** |
| **≥0.1%** | **7** | **2** | **71.4(17.1)** |  | **28.6(18.6)** |  |
|  | | | | | | |
|  | | | | | | |
| **<0.03%** | **106** | **2** | **96.9(1.8)** | **0.018** | **2.2(1.6)** | **0.006** |
| **≥0.03%** | **20** | **3** | **85.0(8.0)** |  | **15.0(8.2)** |  |
|  | | | | | | |
| **Panel D** | | | | | | |
| **MRD** | **n** | **relapses** | **EFS(SE)** | **p** | **CIR(SE)** | **p** |
|  | | | | | | |
| **<0.01%** | **47** | **2** | **93.6(3.6)** | **0.236** | **4.3(3.0)** | **0.147** |
| **≥0.01%** | **30** | **4** | **83.3(6.8)** |  | **13.3(6.3)** |  |
|  | | | | | | |
|  | | | | | | |
| **<0.1%** | **68** | **5** | **89.7(3.7)** | **0.923** | **7.4(3.2)** | **0.701** |
| **≥0.1%** | **9** | **1** | **88.9(10.5)** |  | **11.1(11.1)** |  |
|  | | | | | | |
|  | | | | | | |
| **<0.03%** | **56** | **2** | **94.6(3.0)** | **0.027** | **3.6(2.5)** | **0.024** |
| **≥0.03%** | **21** | **4** | **76.2(9.3)** |  | **19.0(8.8)** |  |
|  | | | | | | |
| **Panel E** | | | | | | |
| **MRD** | **n** | **relapses** | **EFS(SE)** | **p** | **CIR(SE)** | **p** |
|  | | | | | | |
| **<0.01%** | **88** | **4** | **95.4(2.2)** | **0.002** | **4.6(2.2)** | **0.005** |
| **≥0.01%** | **56** | **11** | **77.7(5.7)** |  | **20.5(5.6)** |  |
|  | | | | | | |
|  | | | | | | |
| **<0.1%** | **125** | **8** | **93.5(2.2)** | **<0.001** | **6.5(2.2)** | **<0.001** |
| **≥0.1%** | **19** | **7** | **56.8(11.6)** |  | **37.9(11.9)** |  |
|  | | | | | | |
|  | | | | | | |
| **<0.04%** | **116** | **6** | **94.8(2.1)** | **<0.001** | **5.2(2.1)** | **<0.001** |
| **≥0.04%** | **28** | **9** | **63.9(9.2)** |  | **32.6(9.2)** |  |
|  | | | | | | |
| **Panel F** | | | | | | |
| **MRD** | **n** | **relapses** | **EFS(SE)** | **p** | **CIR(SE)** | **p** |
|  | | | | | | |
| **<0.01%** | **92** | **3** | **93.4(2.6)** | **<0.001** | **3.4(1.9)** | **<0.001** |
| **≥0.01%** | **46** | **14** | **65.3(7.4)** |  | **32.5(7.4)** |  |
|  | | | | | | |
|  | | | | | | |
| **<0.1%** | **122** | **10** | **88.2(3.0)** | **<0.001** | **8.5(2.6)** | **<0.001** |
| **≥0.1%** | **16** | **7** | **55.6(12.6)** |  | **44.4(13.2)** |  |
|  | | | | | | |
|  | | | | | | |
| **<0.04%** | **114** | **8** | **90.1(2.8)** | **<0.001** | **7.3(2.5)** | **<0.001** |
| **≥0.04%** | **24** | **9** | **57.0(10.4)** |  | **38.8(10.6)** |  |

**
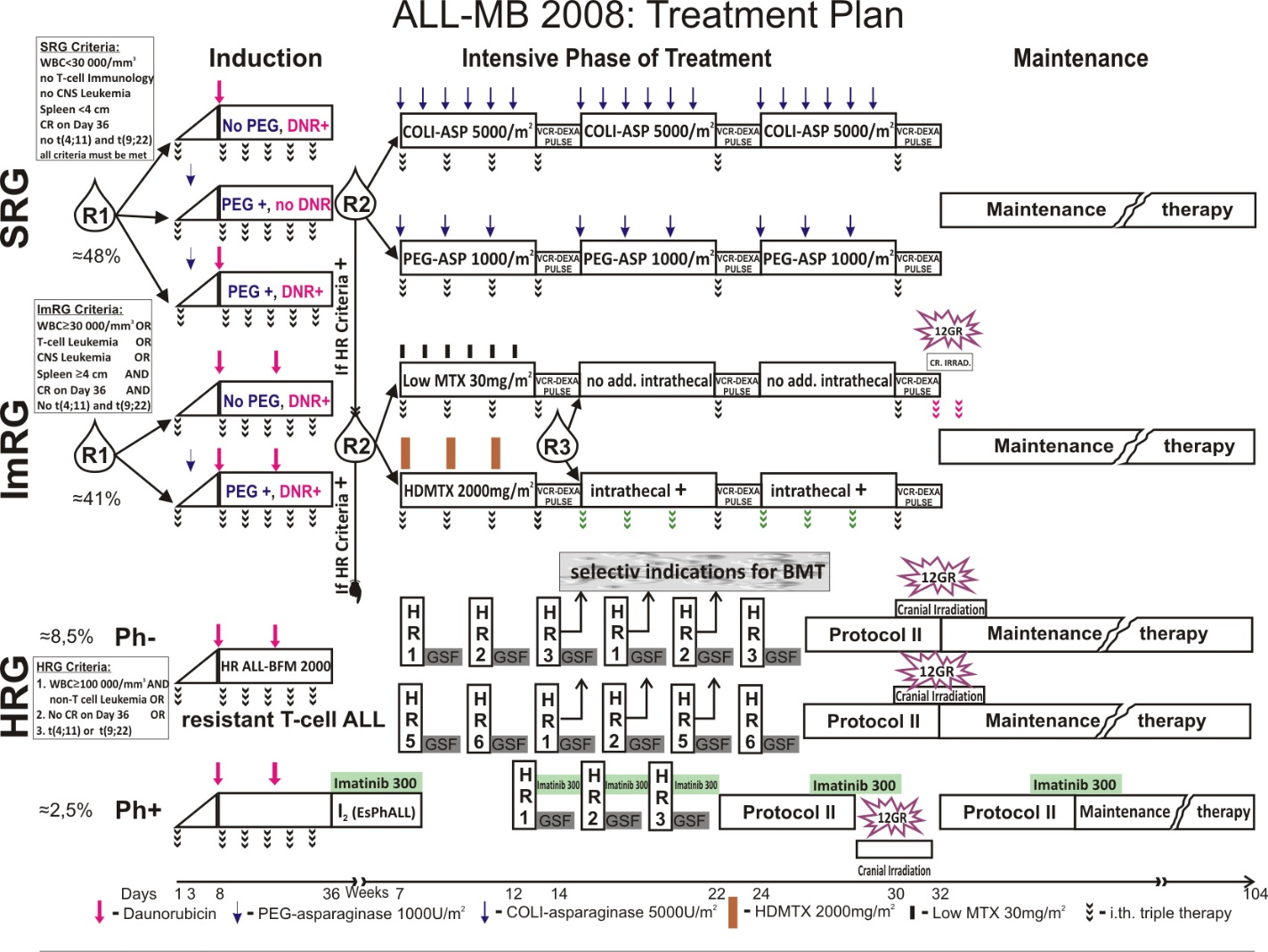
**

**Figure S1.** Principal design of the ALL-MB 2008 protocol

**
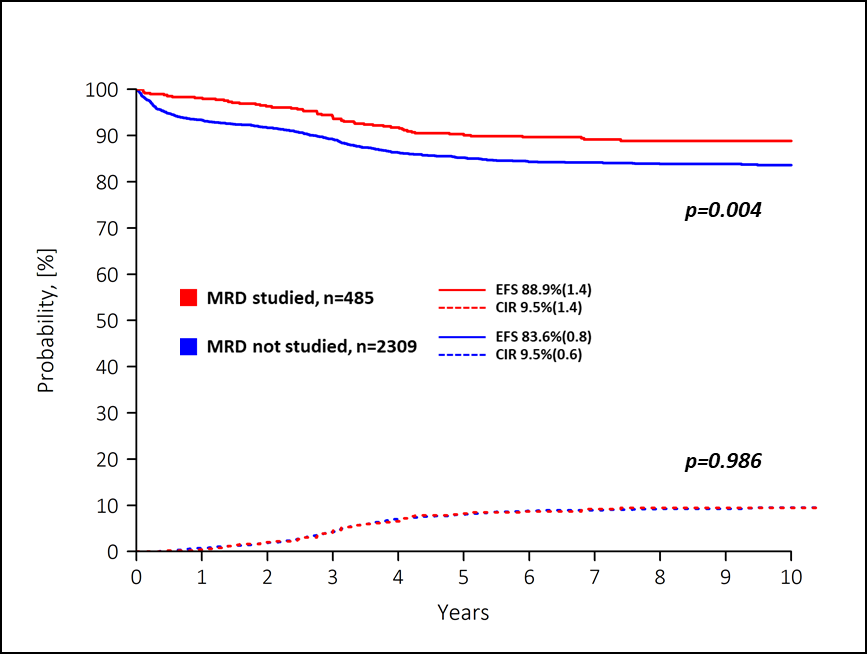
**

**Figure S2.** Event-free survival (EFS, solid lines) and cumulative incidence of relapse (CIR, dashed lines) in patients of the ALL-MB 2008 study with BCP-ALL from the SR and ImR groups without high-risk cytogenetics (n=2794). Patients who were examined for MFC-MRD are shown in blue, the other patients in red. The standard errors are shown in brackets.
